# Supplementary material for: Hypofucosylation promotes pertussis toxin binding to cell surface glycococonjugates and pertussis toxin-induced intracellular ERK signaling
Source: Glycobiology. 2026 Feb 26;36(4):cwag011. doi: 10.1093/glycob/cwag011 (PMC13016894; doi:10.1093/glycob/cwag011)
Supplement: Konada_SupplementaryData_260302 [file konada_supplementarydata_260302.pdf]

Supplementary Data for

Hypofucosylation promotes pertussis toxin binding to cell surface glycococonjugates and pertussis toxin-induced intracellular ERK signaling

Authors: Rohit Sai Reddy Konada, Nicole Nischan, Aurora Silva, Jennifer J. Kohler*

Department of Biochemistry, UT Southwestern Medical Center, Dallas, TX 75390, USA

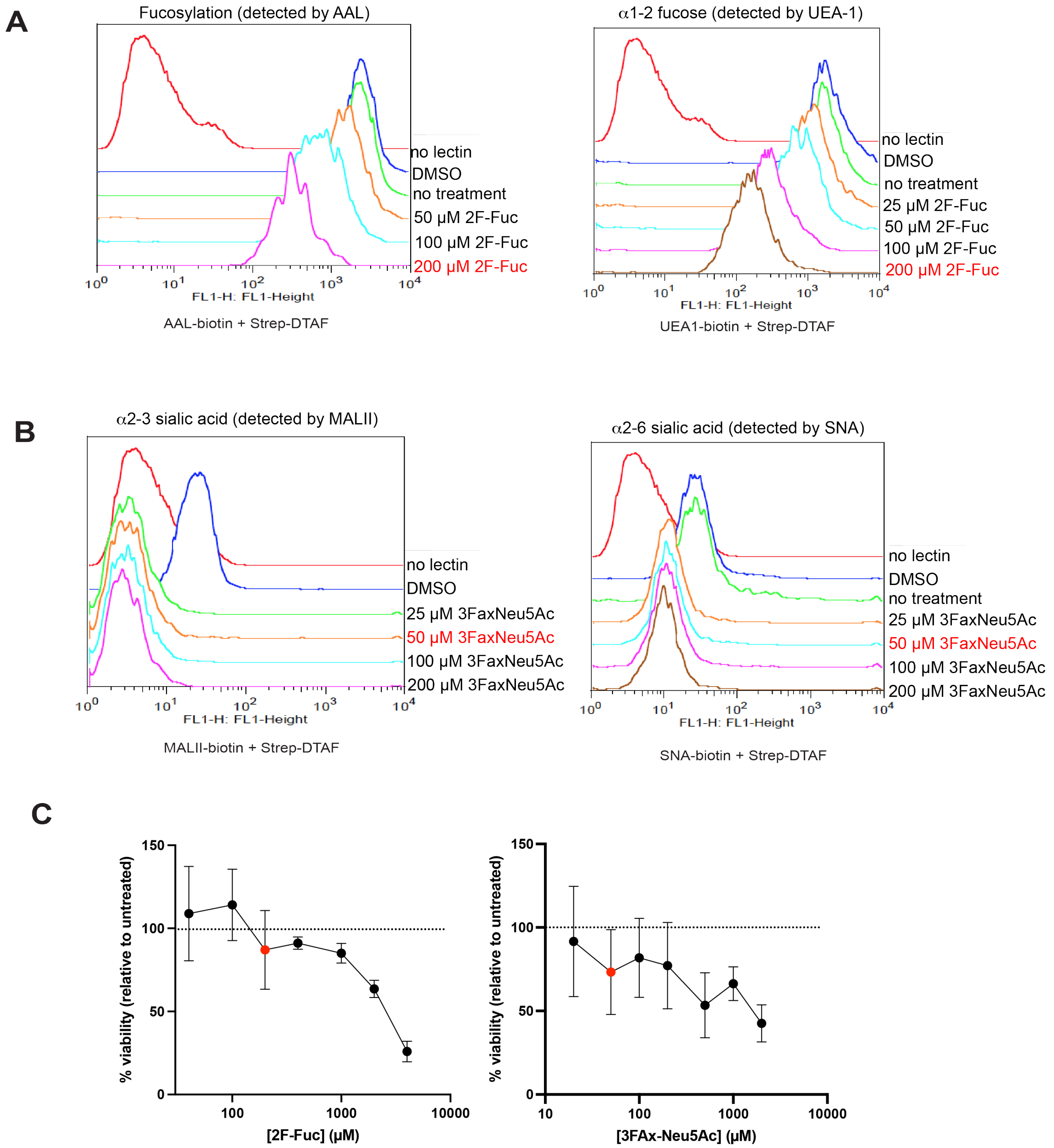

Supplementary Figure 1. Effectiveness and cytotoxicity of small molecule inhibitors of glycosylation in HBEC-3KT cells. HBEC-3KT cells were incubated in media containing a range of glycan inhibitor concentrations to identify the concentration required for successful inhibition of glycan biosynthesis. Cell surface glycans were subsequently analyzed by measuring lectin binding using flow cytometry. **(A)** Flow cytometry histograms showing binding of fucose-specific lectins to HBEC-3KT cell surfaces after culturing with the indicated concentrations of 2-fluorofucose for 72 h. AAL-biotin (left panel) was used to detect multiple forms of fucose and UEA1-biotin (right panel) was used to detect α1-2-linked fucose. Streptavidin-DTAF was used as the secondary detection reagent. **(B)** Flow cytometry histograms showing binding of sialic acid-specific lectins to HBEC-3KT cell surfaces after culturing cells with the indicated concentrations of 3-FaxNeuAc for 72 h. MAL II-biotin (left panel) was used to detect α2-3-linked sialic acid and SNA-biotin (right panel) was used to detect α2-6-linked sialic acid. Streptavidin-DTAF was used as the secondary detection reagent. (**C**) HBEC-3KT cell viability after 72 h incubation with indicated inhibitors. Red symbol indicates the concentration that was selected for experiments.

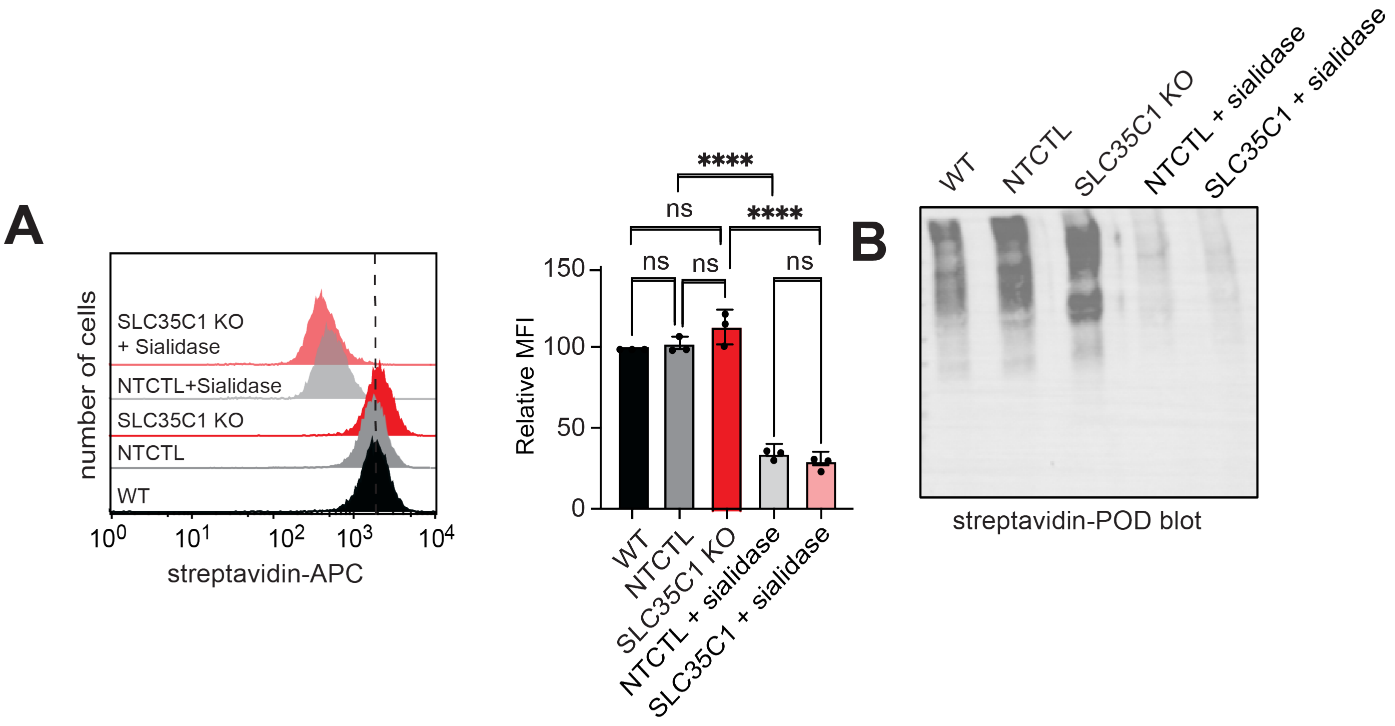

Supplementary Figure 2. Impact of SLC35C1 KO on sialylation. Colo205 cell surface sialic acid was biotinylated by PAL labelling. **(A)** Flow cytometry analysis of PAL-dependent biotinylation of Colo205 cell surfaces. Bar graph shows the quantification of geometric mean fluorescence from 3 independent trials, normalized to the geometric mean of wild type Colo205 cells. **(B)** Streptavidin-POD (peroxidase) blot of lysates from PAL-biotinylated wild type (WT), non-targeted control (NTCTL), and SLC35C1 KO Colo205 cells treated with sialidase or not. Statistical analyses were performed by one-way ANOVA. Error bar indicates mean ± SD, **** indicates $p < 0.0001$; ns $p > 0.05$.

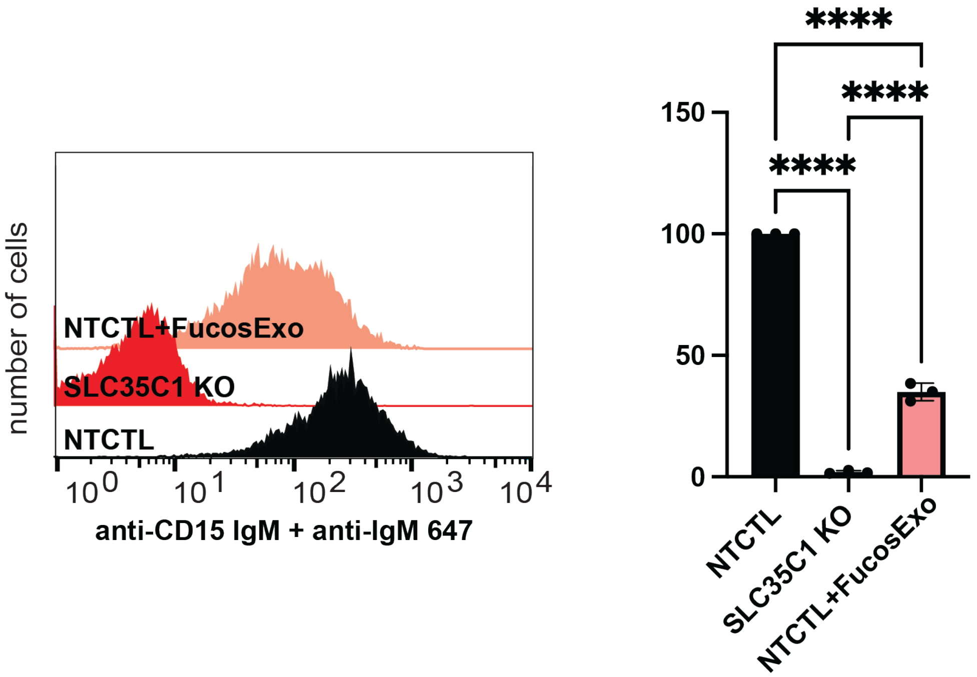

Supplementary Figure 3. Fucosidase treatment reduces Lewis X levels on the surface of Colo205 cells. Flow cytometry histograms showing the binding of anti-Lewis X (anti-CD15) antibody to Colo205 cells. NTCTL (non-targeting control) Colo205 cells were treated with FucosExo™, a mixture of fucosidases, to remove α1-2, α1-3, and α1-4 linked fucose from the cell surface. Fucosidase treatment resulted in a modest reduction in Lewis X as compared to genetic knockout of *SLC35C1*, which eliminated Lewis X expression. The bar graph at right quantifies the results from three replicates. Statistical analyses were performed by one-way ANOVA. Error bar indicates mean ± SD; **** indicates $p < 0.0001$.

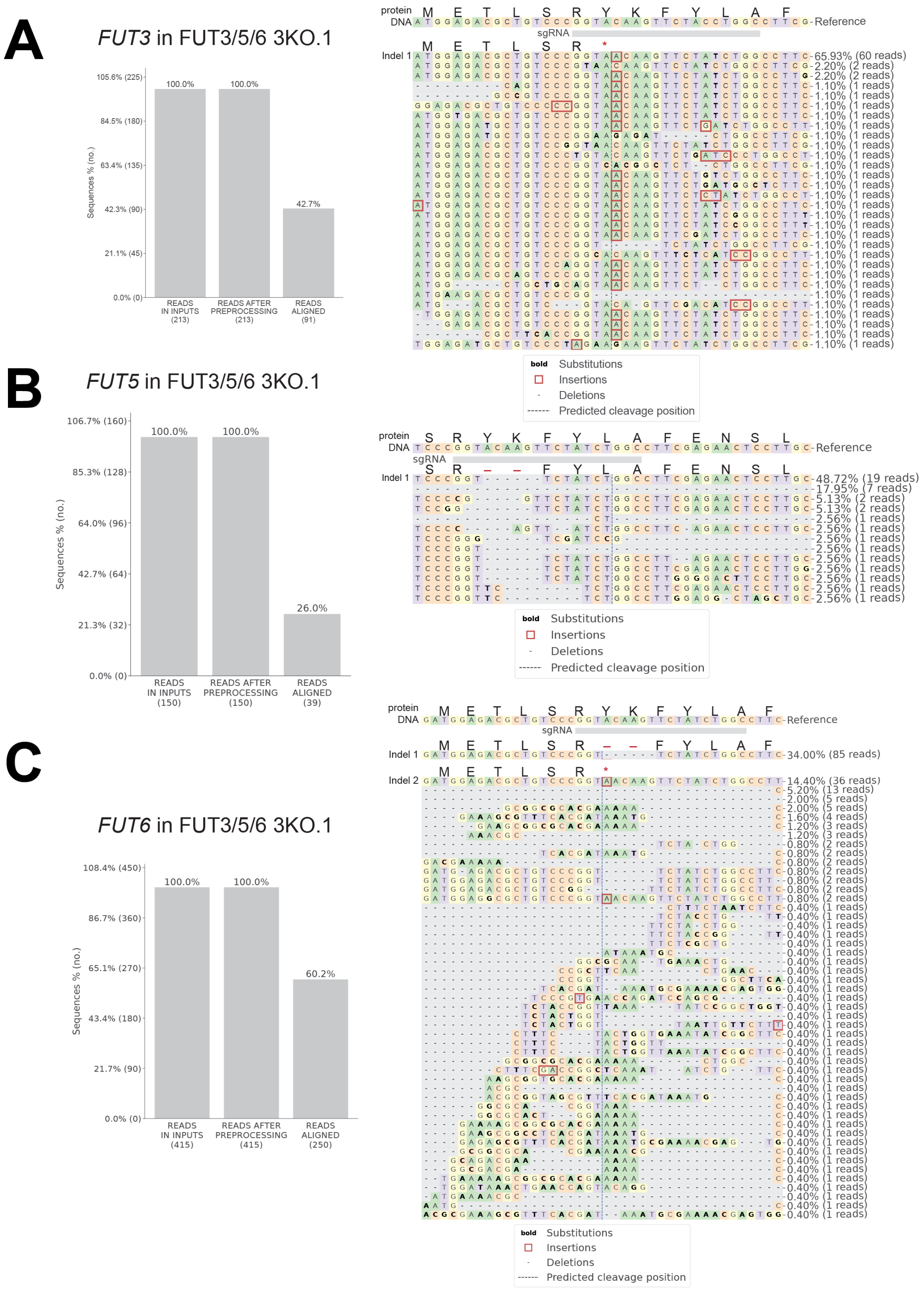

Supplementary Figure 4. Sequence validation of FUT3/5/6 TKO.1 Colo205 cells.

FUT3, *FUT5*, and *FUT6* loci from FUT3/5/6 TKO.1 Colo205 cells were amplified by PCR; the PCR products were sequenced with Oxford nanopore technology. Sequence data were analyzed with CRISPResso2 software. Bar graphs represent the total number of reads and aligned reads. Allele frequency table shows mutations introduced to individual reads. The reference sequence for each cell line shows where the sgRNA binds in the genome. The translated protein sequence is provided for sequences that were detected with more than 15 reads. Detected indels result in a premature stop codon at reside 241 for *FUT3* **(A)**. *FUT5* exhibits the removal of amino acids 254-255 **(B)**. For *FUT6*, indel 1 results in the removal of amino acids 240 and 241, and indel 2 results in a premature stop codon at residue 240 **(C).**

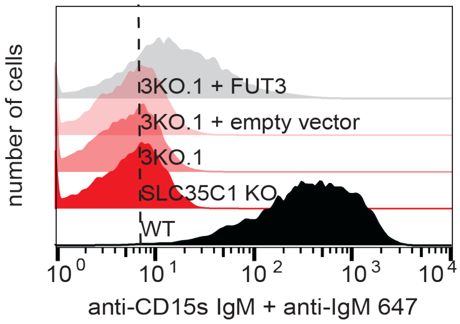

Supplementary Figure 5. Sialyl Lewis X expression on FUT3/5/6 TKO.1 Colo205 cells. FUT3/5/6 TKO.1 Colo205 cells were phenotypically characterized by measuring sialyl Lewis X antibody binding. Flow cytometry histogram showing the binding of mouse anti-human sLewis X (BD Pharmingen™ 551344) to Colo205 cells. Anti-mouse IgM-647 was used as secondary detection reagent.

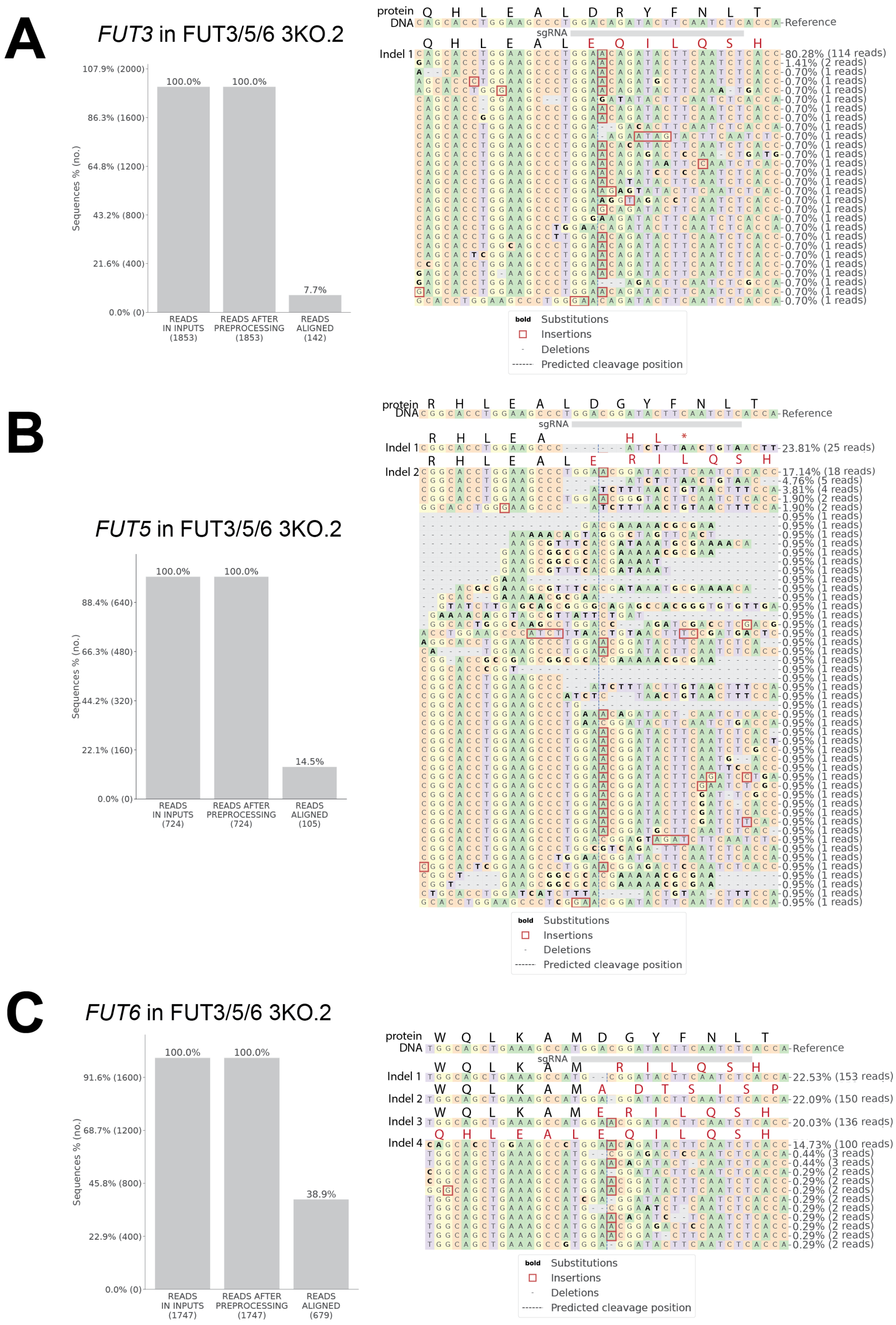

Supplementary Figure 6. Sequence validation of FUT3/5/6 TKO.2 Colo205 cells. *FUT3*, *FUT5*, and *FUT6* loci from FUT3/5/6 TKO.2 Colo205 cells were amplified by PCR; PCR products were sequenced with Oxford nanopore technology. Sequence data were analyzed with CRISPResso2 software. Bar graphs represent the total number of reads and aligned reads. Allele frequency table shows mutations introduced to individual reads. The reference sequence for each cell line shows where the sgRNA binds in the genome. The translated protein sequence is provided for sequences that were detected with more than 15 reads. All detected indels result in a frameshift mutations. These initiate at residue 150 for *FUT3* **(A)**, and at residues 162 and 163 for *FUT5* indels 1 and 2 **(B)**. For *FUT6,* the first three indels initiate at residue 149, while the final indel initiates at 143 **(C)**.

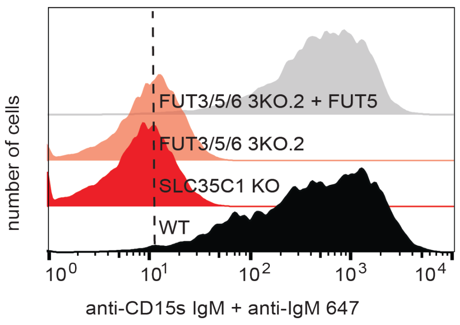

Supplementary Figure 7. Sialyl Lewis X expression on FUT3/5/6 TKO.2 Colo205 cells. FUT3/5/6 TKO.2 Colo205 cells were phenotypically characterized by measuring sialyl Lewis X antibody binding. Flow cytometry histogram showing the binding of mouse anti-human sLewis X (BD Pharmingen™ 551344) to Colo 205 cells. Anti-mouse IgM-647 was used as secondary detection reagent.

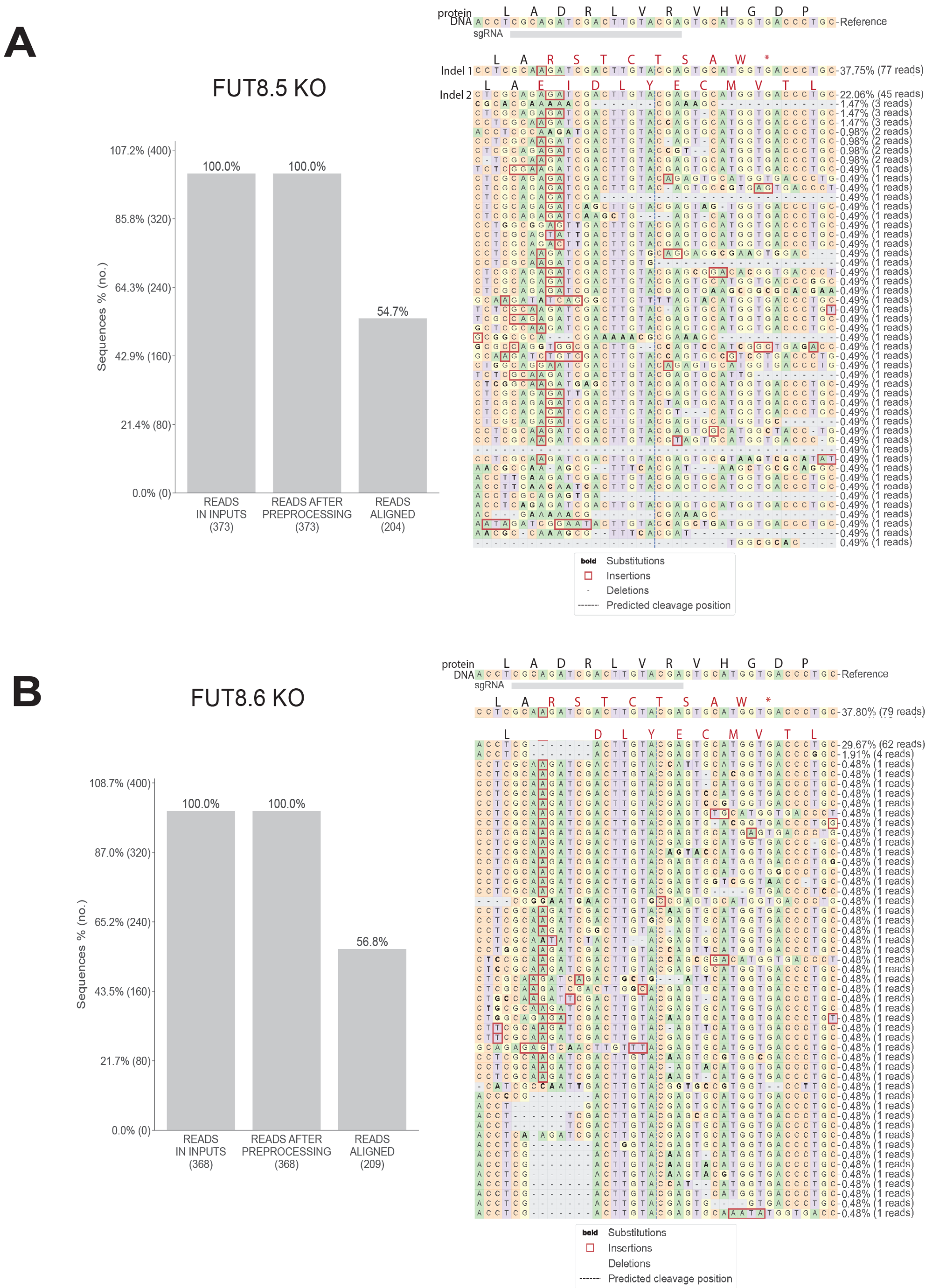

Supplementary Figure 8. Sequence validation of FUT8 KO Colo205 cells. *FUT8* genomic DNA from FUT8 KO Colo205 cell lines was amplified by PCR and the PCR product was sequenced with Oxford nanopore technology. Sequence data were analyzed with CRISPResso2 software. Bar graphs represent the total number of reads and aligned reads. Allele frequency table shows mutations introduced to individual reads. The reference sequence for each cell line shows where the sgRNA binds in the genome. The translated protein sequence is provided for sequences that were detected with more than 15 reads. Both indels for FUT8.5 KO result in frameshift mutations at amino acids 314 **(A)**. Indel 1 for FUT8.6 KO results in a frameshift mutation at amino acid 314, while indel 2 results in a frameshift mutation at amino acid 313 **(B)**. FUT8.5 KO cells were used for all data presented in the main text.

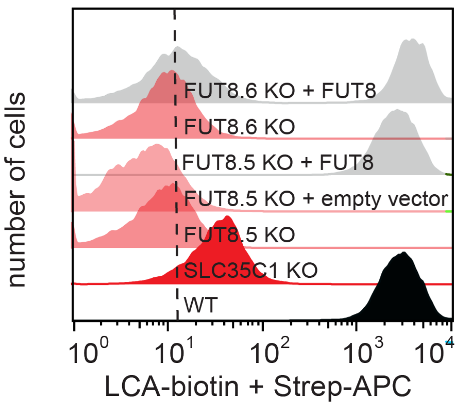

Supplementary Figure 9. Core fucose expression on FUT8 KO Colo205 cells. The FUT8 KO Colo205 cells were phenotypically characterized by measuring LCA binding. Flow cytometry histogram showing the binding of LCA-biotin (Vector labs B-1045-5) to Colo205 cells. Streptavidin-APC (allophycocyanin) was used as secondary detection reagent.

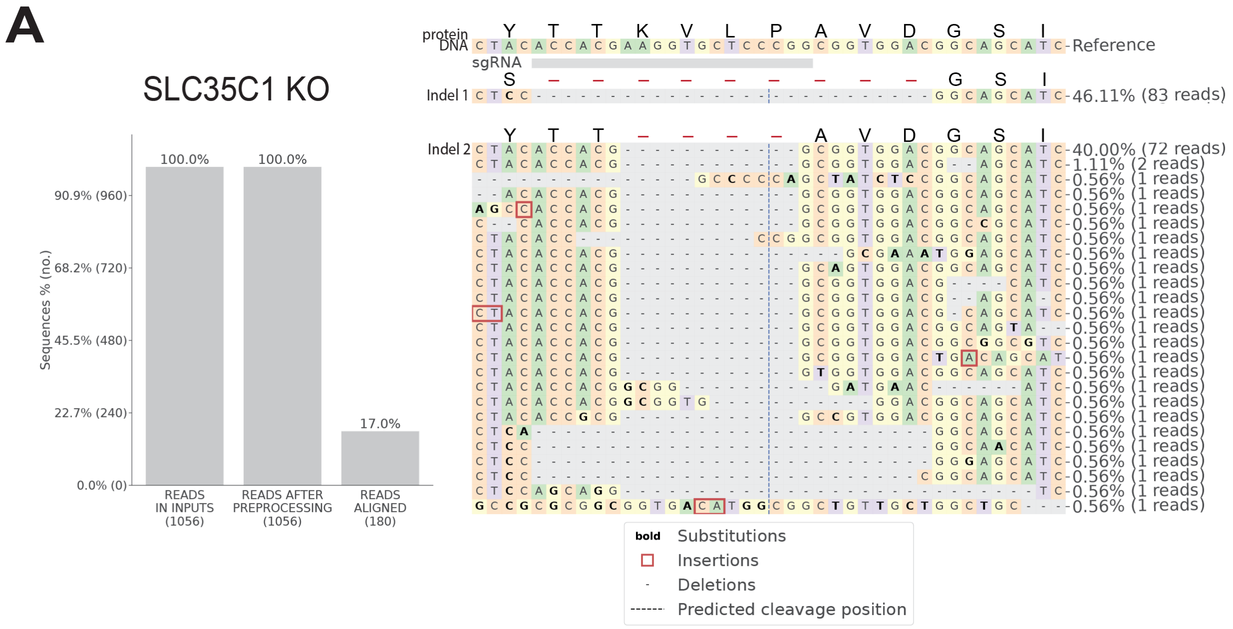

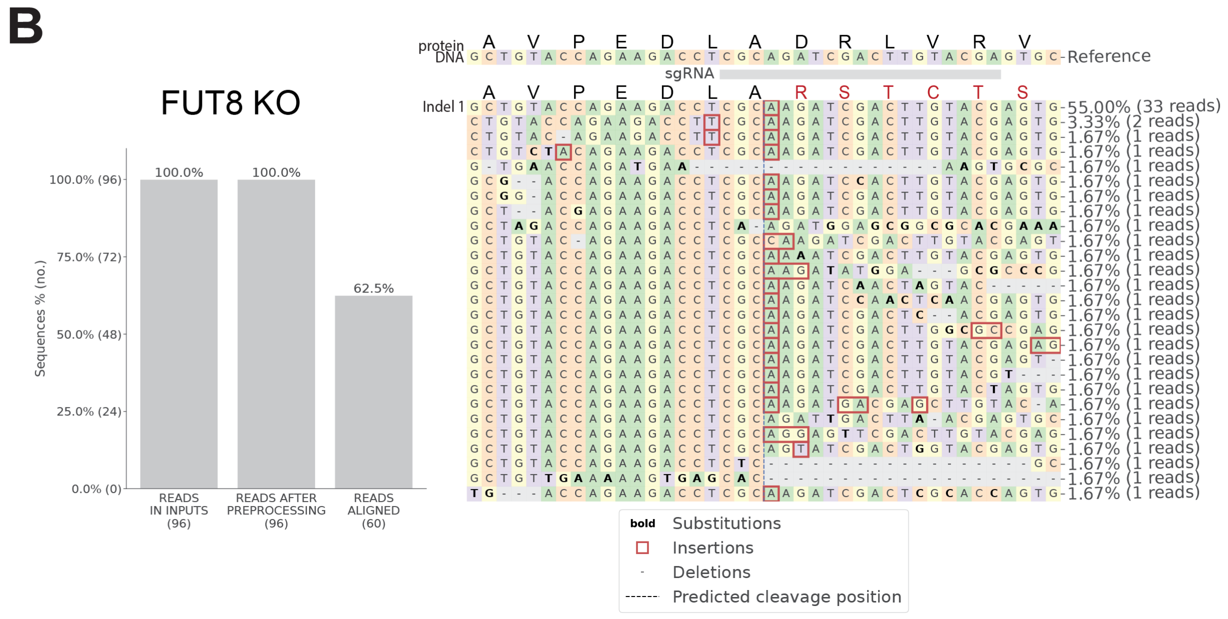

Supplementary Figure 10. Sequence validation of SLC35C1 and FUT8 KO Jurkat cells. *SLC35C1* (**A**) or *FUT8* (**B**) genomic DNA from Jurkat KO cell lines was amplified by PCR and the PCR product was sequenced with Oxford nanopore technology. Sequence data were analyzed with CRISPResso2 software. Bar graphs represent the total number of reads and aligned reads. Allele frequency table shows mutations introduced to individual reads. The reference sequence for each cell line shows where the sgRNA binds in the genome. The translated protein sequence is provided for sequences that were detected with more than 15 reads. Detected indels for *SLC35C1* resulted in the removal of amino acids 216-224 for indel 1, and 218-221 for indel 2 **(A)**. For *FUT8*, a frameshift mutation was introduced at amino acid 314 **(B)**.

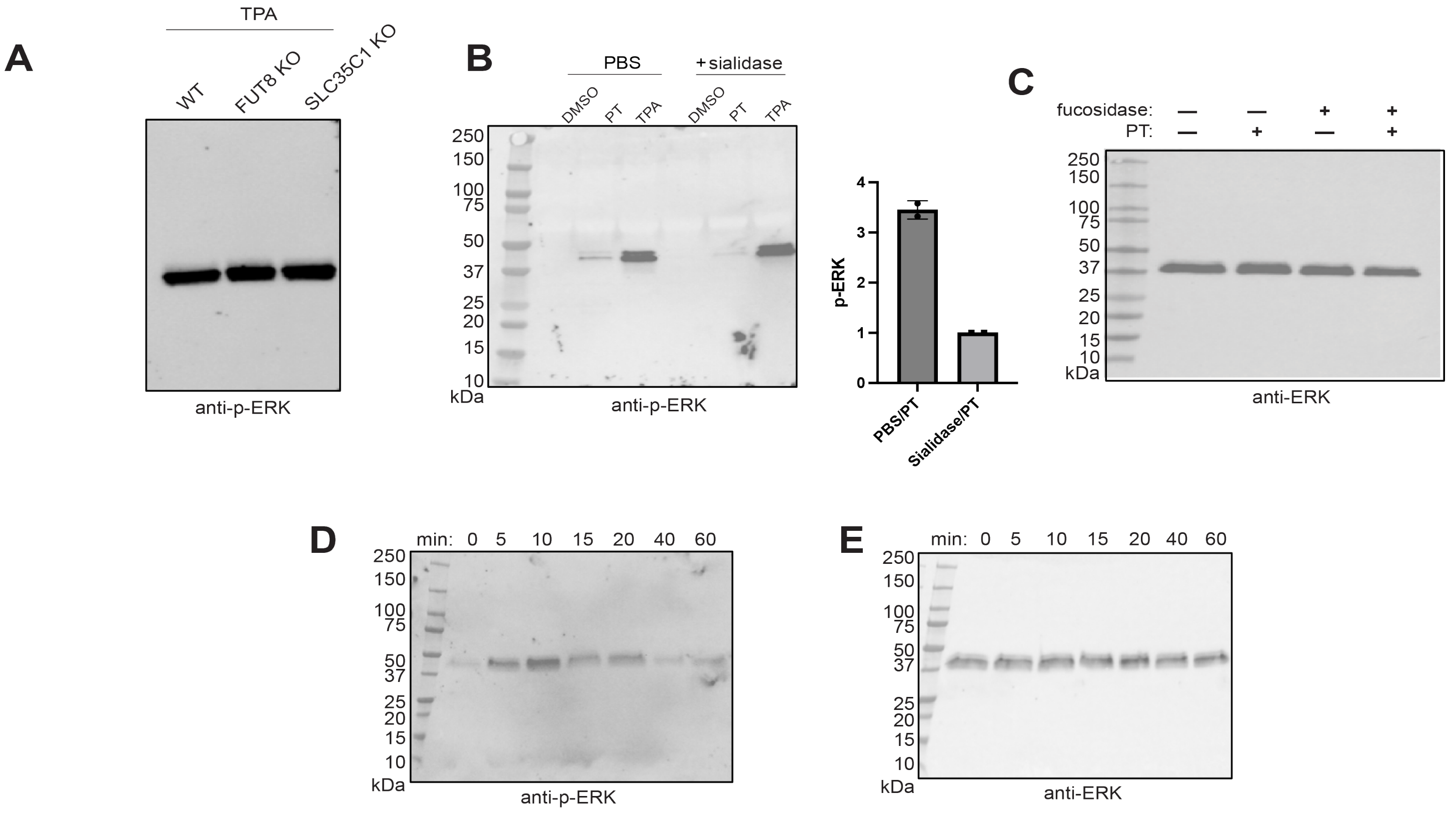

Supplementary Figure 11. ERK phosphorylation in Jurkat cells. (A) Phospho-ERK immunoblot of lysates from TPA-treated (60 min) WT, FUT8 KO and SLC35C1 KO Jurkat cells. **(B)** Phospho-ERK immunoblot of lysates from Jurkat cells with or without sialic acid and induced with either PT or TPA for 60 minutes. Bar graph shows quantification of 2 replicates. **(C)** ERK immunoblot of lysates from PT-induced (60 min) Jurkat cells treated with FucosExo. These data are quantified in Fig. 5D. **(D)** Phospho-ERK immunoblot of lysates from PT-treated Jurkat cells at different time points, ranging from 0 to 60 minutes. Single replicate. **(E)** ERK immunoblot of lysates from PT-treated Jurkat cells at different time points, ranging from 0-60 minutes. Single replicate.
